# Supplementary material for: Real time monitoring of respiratory viral infections in cohort studies using a smartphone app
Source: iScience. 2024 Sep 17;27(10):110912. doi: 10.1016/j.isci.2024.110912 (PMC11439530; doi:10.1016/j.isci.2024.110912)
Supplement: Document S1. Figure S1, Tables S1, and S2 [file mmc1.pdf]

iScience, Volume *27*

Supplemental information

Real time monitoring of respiratory viral infections in cohort studies using a smartphone app

David G. Hancock, Elizabeth Kicic-Starcevich, Thijs Sondag, Rael Rivers, Kate McGee, Yuliya V. Karpievitch, Nina D'Vaz, Patricia Agudelo-Romero, Jose A. Caparros-Martin, Thomas Iosifidis, Anthony Kicic, and Stephen M. Stick

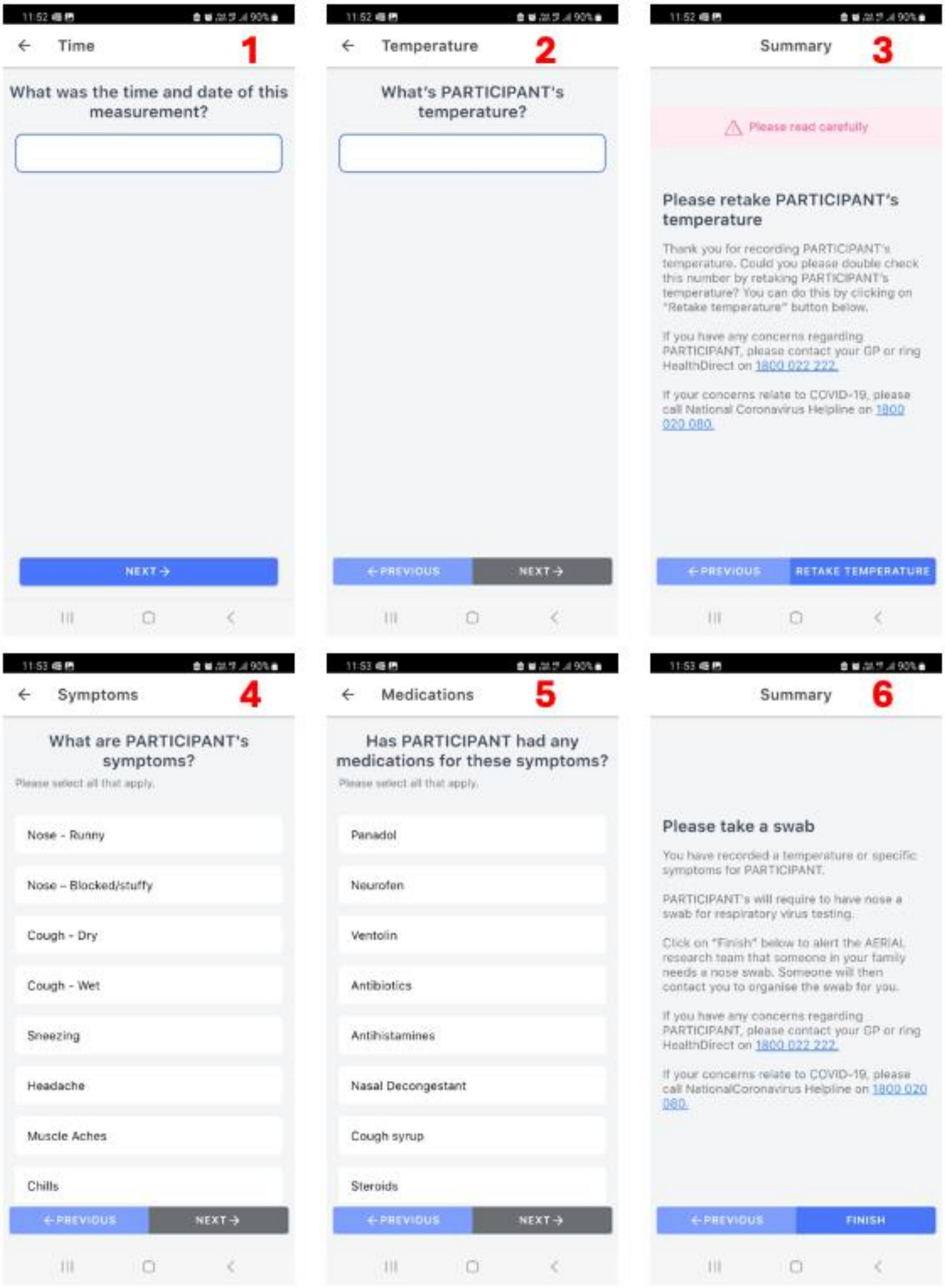

Figure S1. TempTracker App. Participant's (or the parents of participants) entered the following data into the app: (1) the time/date and (2) the value of daily temperature measurements. (3) Participants were prompted to recheck and record temperatures >37.5. Participants were then asked to record (4) symptoms and (5) medications from a pre-defined list, with option to report other symptoms/medications in a free-text box. (6) Events meeting the case definition for a symptomatic respiratory illness generated an automated "Please take a swab" alert to participants and study staff.

Table S1: Selected responses from consumers during testing of prototype.

Supportive feedback	Feedback suggesting improvement
I deliberately put a high temp- the prompts to swab and instructions worked really well.	When you fill out a high temp and it asks you to enter it again it would be easier if you didn't have to refill the symptoms again. Not sure if that is possible. just seems a bit onerous when you have a sick child.
Looks smart but also simple and easy to use	I don't think it should be as easy to add a temperature from a past date. When looking at the screen if you are in a previous date it looks no different from todays view and it is easy to add a temperature reading without realising you are on the wrong day.
Great aerial app, easy to use, good clear explanations.	Perhaps a second reminder to take temp that day. If you are busy when the first reminder comes up you can't snooze it and no further reminders are given.
Was able to put all ID on many devices without a problem	When phone is on powersave mode, it does not prompt to take children's temp. I was only notified at 10pm when I plugged in the phone to charge before bed.
If no temps or symptoms have been recorded a reminder prompt is sent out	Download not the easiest – but I have a new phone so this probably didn't help.

Table S2: List of all symptoms and medications included in the TempTracker app. Respiratory symptoms for case definition denoted with *.

SYMPTOMS	MEDICATIONS
NONE	NONE
Nose – Runny*	Panadol
Nose – Blocked/stuffy*	Neurofen
Cough – Dry*	Ventolin
Cough – Wet*	Antibiotics
Sneezing*	Antihistamines
Headache*	Nasal Decongestant
Muscle Aches*	Cough syrup
Chills*	Steroids
Repeated shaking with chills*	Other -> Free Text Entry
Tiredness*	
Sore Throat*	
Having trouble breathing*	
New loss of taste*	
New loss of smells*	
Feeding – not wanting*	
Not eating solids*	
Diarrhoea	
Crying	
Sleeping more	
Feeds – wanting more	
Cling	
Dribbling	
Rubbing at gums	
Pulling at ears	
Vomiting	
Nappies – less wet	
Nappies – more wet	
Rash	
Flushed cheeks/face	
Wind/colic	
Other -> Free Text Entry	
